# Patterns and prognostic implications of distant metastasis in breast Cancer based on SEER population data

**DOI:** 10.1038/s41598-025-12883-x

**Published:** 2025-07-23

**Authors:** Maoquan zhang, Hanqing Deng, Rihua Hu, Fuwei Chen, Shiwen Dong, Shiyi Zhang, Wenyan Guo, Wen Yang, Wenxin Chen

**Affiliations:** 1 Department of Breast Surgery, Sanming First Hospital Affiliated to Fujian Medical University, 29 Liedong Road, Sanming, 365001 Fujian China; 2 Department of Clinical Medicine, Fujian Medical University, Fuzhou, 350001 Fujian China; 3 Department of Surgery, Datian General Hospital, Sanming, 366100 Fujian China

**Keywords:** Breast cancer, Distant metastasis, SEER database, Molecular subtype, Multi-organ metastasis, Prognosis, Survival analysis, Clinicopathological features, Diseases, Medical research, Oncology, Risk factors

## Abstract

Distant metastasis remains the leading cause of mortality in breast cancer, yet comprehensive population-based evaluations of metastatic site combinations and their survival implications are limited. This study aimed to explore the clinicopathological determinants and prognostic outcomes of site-specific and multi-organ metastases in breast cancer using SEER data. A total of 200,558 female breast cancer patients diagnosed between 2014 and 2023 were extracted from the SEER database. Logistic regression was used to assess associations between clinicopathological features and metastatic patterns. Kaplan–Meier analysis and Cox proportional hazards models were applied to evaluate overall survival (OS) across different metastatic site combinations. Among patients with distant metastasis classified into 15 common metastatic patterns, bone was the most common metastatic site (21.3%), followed by lung (16.1%), liver (9.2%), and brain (2.9%). Molecular subtypes showed distinct organotropism: HR+/HER2 − tumors were prone to bone-only metastasis, whereas HER2-positive and triple-negative subtypes were more likely to involve visceral and brain metastases. Multi-organ metastases, especially combinations including the brain (e.g., brain + liver + lung), were associated with the poorest prognosis (median OS: 4.0 months). Younger age (≤ 40 years), higher histological grade (Grade III), and tumor location in the axillary tail or unspecified regions were independently associated with increased metastatic risk. Grade III tumors exhibited broader visceral spread and significantly worse survival compared to lower-grade tumors. This is the first population-based study to systematically characterize 15 metastatic site combinations and their survival outcomes across molecular subtypes. The findings highlight the heterogeneity of breast cancer metastasis and underscore the need for subtype-specific, site-targeted surveillance strategies and prognostic assessment tools.

## Introduction

Breast cancer is the most common malignancy in women and a leading cause of cancer-related death worldwide, with over 2.3 million new cases and 685,000 deaths in 2020 alone^1^. Despite significant advances in screening, diagnosis, and systemic therapies, distant metastasis remains the predominant cause of mortality in breast cancer patients^2^. Common metastatic sites include bone, liver, lung, and brain, and the presence of distant metastasis is associated with significantly reduced overall survival (OS) and quality of life^3^.

Previous studies have shown that the pattern of metastatic spread is influenced by clinicopathological and molecular characteristics of the primary tumor. Hormone receptor (HR)-positive breast cancers tend to metastasize to bone, whereas HER2-positive and triple-negative breast cancers (TNBC) are more prone to visceral metastases such as liver, lung, or brain^4,5^. However, most of these studies have focused on single-organ metastasis or specific molecular subtypes, and comprehensive analyses comparing single- and multi-organ metastasis across different subgroups remain limited^6–8^.

Moreover, while the prognostic significance of metastasis to individual organs (e.g., brain vs. bone) has been explored, the impact of multi-organ metastases (i.e., involvement of two or more sites) on survival outcomes is still controversial. Some studies suggest that the number of metastatic sites is an independent predictor of poor prognosis^9^, while others argue that molecular subtype may play a more decisive role regardless of the extent of metastasis^10^. This discrepancy highlights the need for a more detailed and stratified analysis.

Another important but underexplored aspect is how clinicopathological factors such as age, tumor location, histological grade, and molecular subtype influence not only metastatic patterns but also the risk of multi-organ spread, and whether this interaction contributes differently to survival outcomes. Furthermore, real-world evidence from large population-based datasets remains scarce, especially regarding how these factors interact to predict survival in patients with single-organ vs. multi-organ metastasis.

To address these knowledge gaps, the present study aims to:

- Examine the associations between specific sites of distant metastasis (bone, liver, lung, and brain) and clinicopathological features, including age at diagnosis, tumor location, histologic grade, pathological classification, and molecular subtype;
- Compare survival outcomes across patients with single-organ, dual-organ, and multi-organ (≥ 3 sites) metastases, and evaluate how these outcomes vary by molecular subtype;
- Identify prognostic factors independently associated with multi-organ metastasis and assess their impact on overall survival through multivariate regression models;
- Provide real-world, population-level insights based on the Surveillance, Epidemiology, and End Results (SEER) database, a comprehensive and high-quality registry of breast cancer patients in the United States.

This study seeks to enhance the current understanding of metastatic heterogeneity and its prognostic implications in breast cancer, with the goal of informing individualized treatment decisions, follow-up strategies, and risk stratification approaches in metastatic settings.

## Materials and methods

### Data source and study population

This retrospective cohort study utilized data from the Surveillance, Epidemiology, and End Results (SEER) 18 registries, which collectively cover approximately 28% of the U.S. population. Female patients diagnosed with primary breast cancer between January 2014 and November 2023 were identified using SEER*Stat software. All included cases were confirmed histologically.

**Fig. 1:**
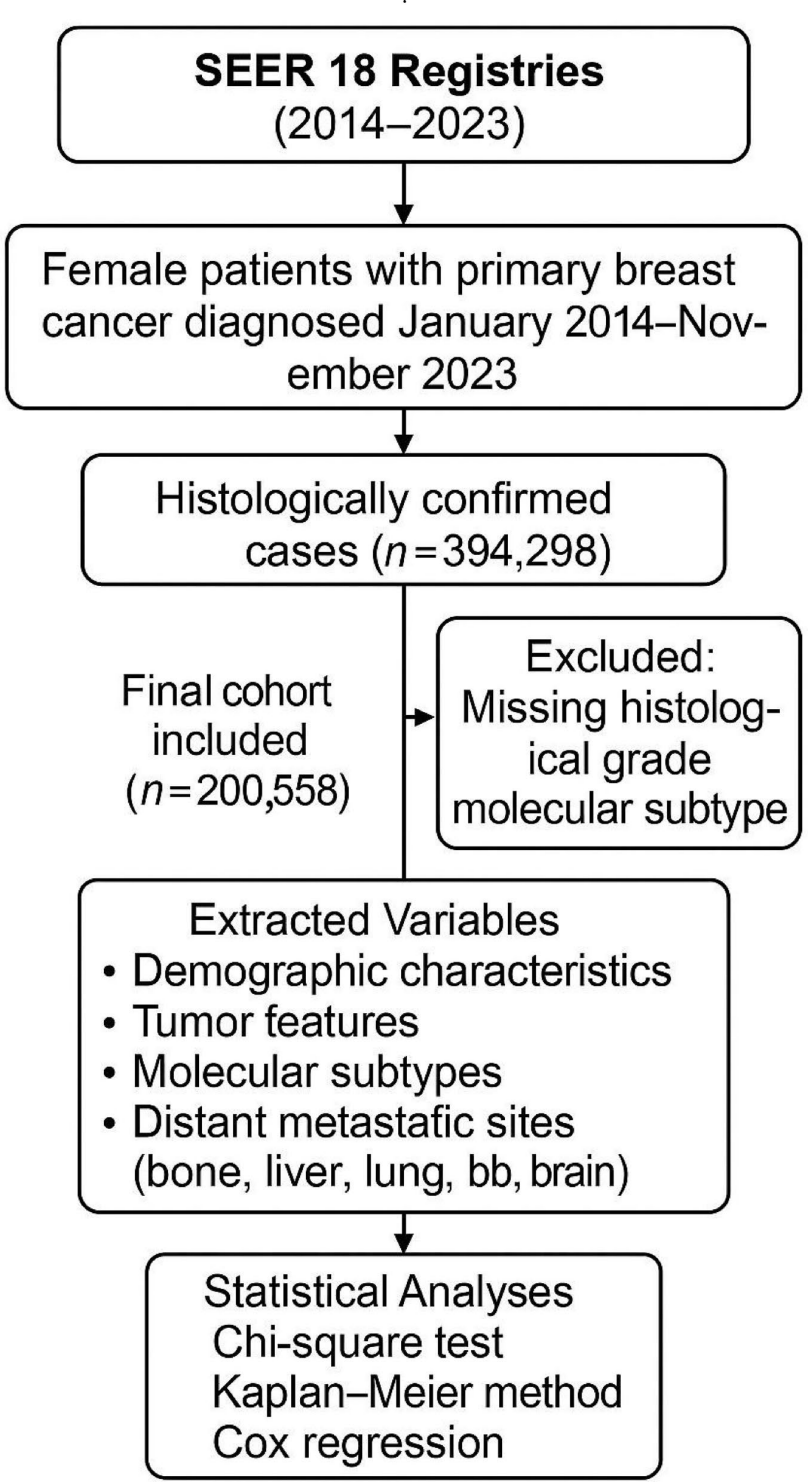
Flowchart of study design and patient selection. Flow diagram illustrating the overall study design, patient inclusion and exclusion criteria, and analytical steps based on SEER data collected from 2014 to 2023.

 A total of 394,298 female patients diagnosed with primary breast cancer were initially identified from the SEER database. Cases with missing or incomplete data on key clinical and pathological variables—including estrogen receptor (ER), progesterone receptor (PR), HER2 status, histological grade, metastatic status at diagnosis, and survival time—were excluded. After applying these exclusion criteria, 200,558 patients were included in the final analysis (Fig. 1).

### Variable definitions

#### Demographic characteristics

Age at diagnosis was categorized into eight groups: 18–29, 30–39, 40–49, 50–59, 60–69, 70–79, 80–84, and ≥ 85 years.

#### Tumor characteristics

Tumor-related variables included:

- Primary site: Specific anatomical location within the breast (e.g., upper-outer quadrant, central portion).
- Laterality: Tumor side (left or right breast).
- Histological type: Classified using ICD-O-3 codes and grouped into major categories (e.g., ductal and lobular neoplasms).
- Histological grade: Reported as Grade I, II, or III.
- Clinical TNM staging: Including tumor size (T), nodal status (N), metastasis (M), and AJCC stage groupings (e.g., Stage I–IV).
- Molecular Subtypes: Molecular subtypes were defined based on immunohistochemical expression of estrogen receptor (ER), progesterone receptor (PR), and human epidermal growth factor receptor 2 (HER2), categorized as follows:HR+/HER2−；HR+/HER2+；HR−/HER2+；Triple-negative breast cancer (TNBC: ER−, PR−, HER2−).

#### Sites of distant metastasis

Distant metastases to bone, liver, lung, and brain were recorded at the time of initial diagnosis. Patients were further grouped based on the number of metastatic sites: single-organ, two-organ, and multi-organ (≥ 3) involvement.

According to SEER definitions, only metastases present at initial diagnosis and recorded under the M1 classification were included. Regional recurrences—including chest wall, ipsilateral axillary, supraclavicular, and internal mammary lymph node involvement—were not considered distant metastases and were excluded. In contrast, distant lymph node metastases (e.g., cervical or contralateral axillary nodes), if documented as M1, were classified and analyzed as distant metastasis along with visceral organs.

#### Survival outcomes

Overall survival (OS) was defined as the time (in months) from diagnosis to death from any cause or last follow-up. Survival status was determined using the variable “Vital status recode” (Alive or Dead).

### Statistical analysis

All statistical analyses were conducted using R software (version 4.3.2) and SPSS (version 27.0; IBM Corp., Armonk, NY, USA). Descriptive statistics were used to summarize baseline patient characteristics. Categorical variables were presented as frequencies and percentages.

Chi-square tests were employed to assess associations between clinicopathological features and the presence or distribution of distant metastases. Multivariable logistic regression models were constructed to estimate adjusted odds ratios (ORs) and 95% confidence intervals (CIs) for site-specific metastasis by age group, histological type, grade, and tumor location. The 41–60 years age group served as the reference category in age-based models.

The distribution patterns of metastatic combinations were evaluated across different molecular subtypes using cross-tabulation and proportion plots. Fifteen distinct metastatic patterns were selected based on frequency, including single-organ, dual-organ, triple-organ, and quadruple-organ (bone, liver, lung, and brain) involvement. Proportions were calculated relative to the total number of patients exhibiting these selected patterns, ensuring that the sum equaled 100%. Organotropism was assessed by comparing the relative frequencies of each breast cancer subtype across individual and combined metastatic sites.

Survival analysis was performed using the Kaplan–Meier method, with log-rank tests used to compare survival curves among groups stratified by metastatic site and burden (single, double, triple, and quadruple organ involvement). Median survival times were calculated for each group. A two-tailed p-value < 0.05 was considered statistically significant.

## Results

### Baseline characteristics of the study population

A total of 200,558 female breast cancer patients diagnosed between January 2014 and November 2023 were included. The most common age group was 65–69 years (14.5%), followed by 60–64 (13.8%) and 70–74 (12.6%). Most tumors originated in the upper-outer quadrant (34.5%), with laterality nearly balanced between the left (50.8%) and right (49.1%) sides.

Grade II tumors were the most frequent (47.0%), followed by Grade I (26.9%) and Grade III (26.0%). The predominant histological subtype was ductal/lobular carcinoma (97.4%). Regarding TNM staging, T1c (34.2%) and T2 (31.6%) tumors were most common. Most patients had no nodal involvement (N0, 61.6%) or distant metastasis at diagnosis (M0, 95.2%).

In molecular subtyping, HR+/HER2 − tumors were predominant (76.0%), followed by HR+/HER2+ (10.1%), triple-negative (10.0%), and HR−/HER2+ (3.8%). Among metastatic cases, bone was the most frequent site (3.1%), followed by lung (1.4%), liver (1.1%), and brain (0.3%). At last follow-up, 92.4% of patients were alive.(Table 1).

**Table 1:** Summary of baseline characteristics (*n* = 200,558).

Variable	*n* (%)	Variable	*n* (%)
Age group		Molecular subtype	
<40	11,820 (5.9%)	HR+/HER2−	152,434 (76.0%)
40–49	36,214 (18.1%)	HR+/HER2+	20,333 (10.1%)
50–59	48,017 (23.9%)	TNBC	20,058 (10.0%)
60–69	56,778 (28.3%)	HR−/HER2+	7,631 (3.8%)
≥70	47,729 (23.8%)	Metastatic site	
Primary site		Bone	6,218 (3.1%)
Upper-outer quadrant	69,228 (34.5%)	Lung	2,808 (1.4%)
Other	131,330 (65.5%)	Liver	2,194 (1.1%)
Laterality		Brain	622 (0.3%)
Left	101,872 (50.8%)	Vital status	
Right	98,461 (49.1%)	Alive	185,276 (92.4%)
Grade		Dead	15,282 (7.6%)
I	53,960 (26.9%)		
II	94,279 (47.0%)		
III	52,121 (26.0%)		

### Factors associated with Site-Specific metastasis

**Fig. 2:**
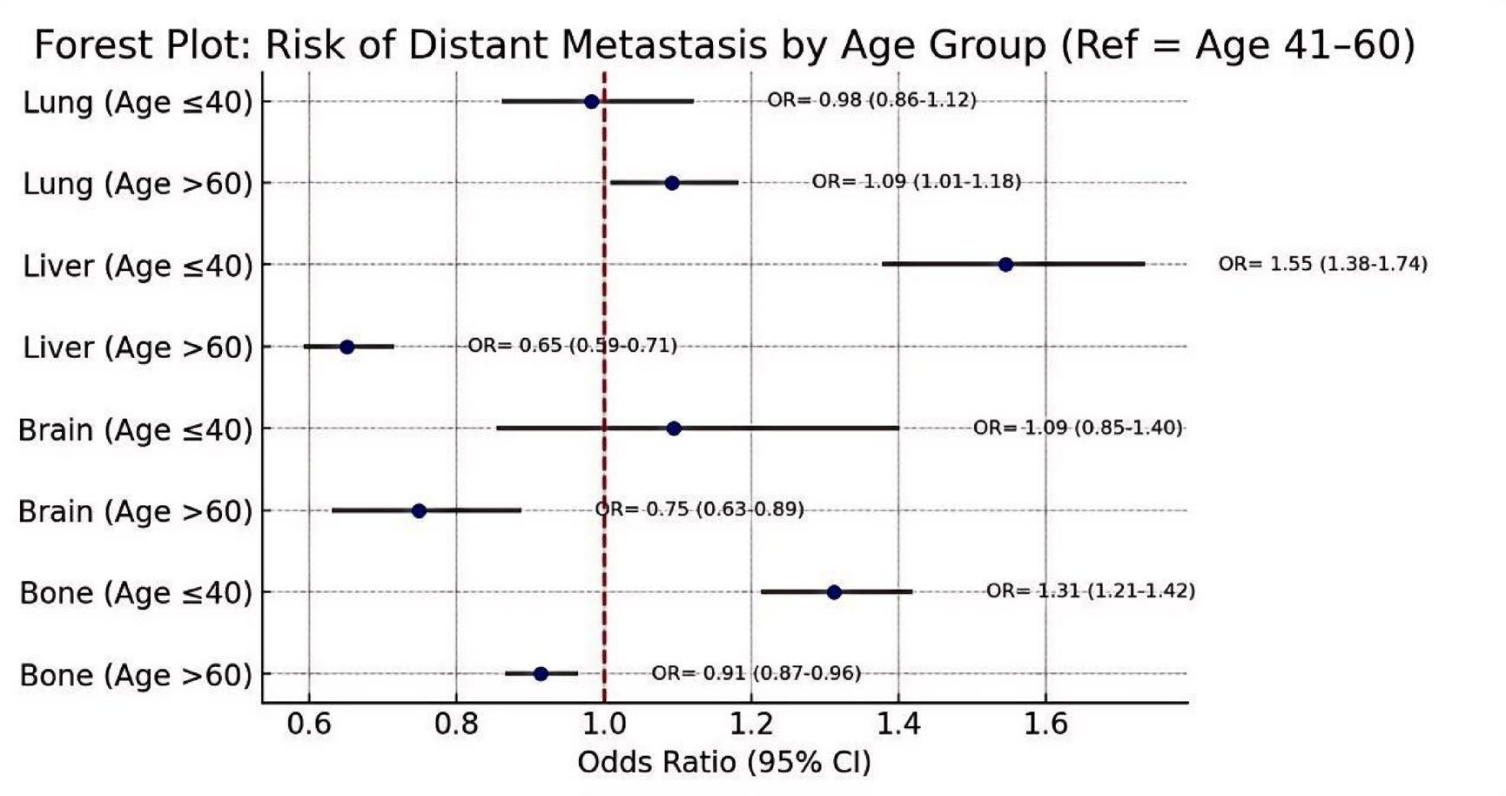
Forest plot of adjusted odds ratios for site-specific distant metastasis by age group. Forest plot showing the adjusted odds ratios (ORs) and 95% confidence intervals (CIs) for site-specific distant metastases across age groups, using patients aged 41–60 years as the reference. Estimates were derived from multivariate logistic regression analysis.

Multivariate logistic regression revealed that age, histological grade, and tumor location were significantly associated with variations in distant metastatic risk across organ sites (Table 2). Patients aged ≤ 40 years had increased risks of bone (OR = 1.31) and liver metastases (OR = 1.55), while those > 60 years showed decreased risks of bone (OR = 0.91), liver (OR = 0.65), and brain (OR = 0.75) metastases, compared to the 41–60-year reference group (Fig. 2). No significant difference was observed for lung metastases.The age group of 41–60 years was chosen as the reference category because it represents the most prevalent age range for breast cancer diagnosis and provides a stable clinical baseline for comparing the metastatic risks associated with both younger (≤ 40 years) and older (> 60 years) patients.

**Table 2:** Adjusted odds ratios (ORs) and 95% confidence intervals (CIs) for site-specific distant metastases by clinical and pathological factors.

Variable	Bone OR (95% CI)	Liver OR (95% CI)	Lung OR (95% CI)	Brain OR (95% CI)
Age ≤ 40 vs. 41–60	1.55 (1.38–1.74)	1.55 (1.38–1.74)	0.98 (0.86–1.12)	1.09 (0.85–1.40)
Age > 60 vs. 41–60	0.91 (0.87–0.96)	0.65 (0.59–0.71)	1.09 (1.01–1.18)	0.75 (0.63–0.89)
Grade III vs. Grade I	1.22 (1.13–1.33)	1.60 (1.38–1.85)	1.36 (1.22–1.52)	1.71 (1.29–2.28)
Grade II vs. Grade I	1.10 (1.03–1.18)	1.23 (1.08–1.41)	1.14 (1.03–1.27)	1.34 (1.03–1.76)
Axillary tail vs. UOQ	1.35 (1.16–1.56)	1.48 (1.16–1.88)	1.20 (0.97–1.48)	1.46 (0.93–2.28)
Unspecified site vs. UOQ	1.42 (1.30–1.56)	1.61 (1.39–1.87)	1.33 (1.16–1.52)	1.57 (1.19–2.06)

Tumor location significantly influenced metastatic potential. Compared to tumors located in the upper-outer quadrant (UOQ), those in the axillary tail (C50.6) showed increased risks of bone (OR = 1.35, 95% CI: 1.16–1.56), liver (OR = 1.48, 95% CI: 1.16–1.88), and brain metastases (OR = 1.46, 95% CI: 0.93–2.28), although the latter was not statistically significant. Tumors in unspecified locations (C50.9) were associated with even higher risks across all sites, including bone (OR = 1.42), liver (OR = 1.61), lung (OR = 1.33), and brain (OR = 1.57), indicating a strong association with multi-organ spread.

Histological grade also played an important role in determining metastatic behavior. Compared to Grade I tumors, Grade II and III tumors exhibited progressively higher risks of visceral and brain metastases. Grade III tumors, in particular, were significantly associated with liver (OR = 1.60), lung (OR = 1.36), and brain (OR = 1.71) metastases, highlighting the broader metastatic spectrum and biological aggressiveness of poorly differentiated tumors.

### Molecular subtypes and Organ-Specific metastasis

**Fig. 3:**
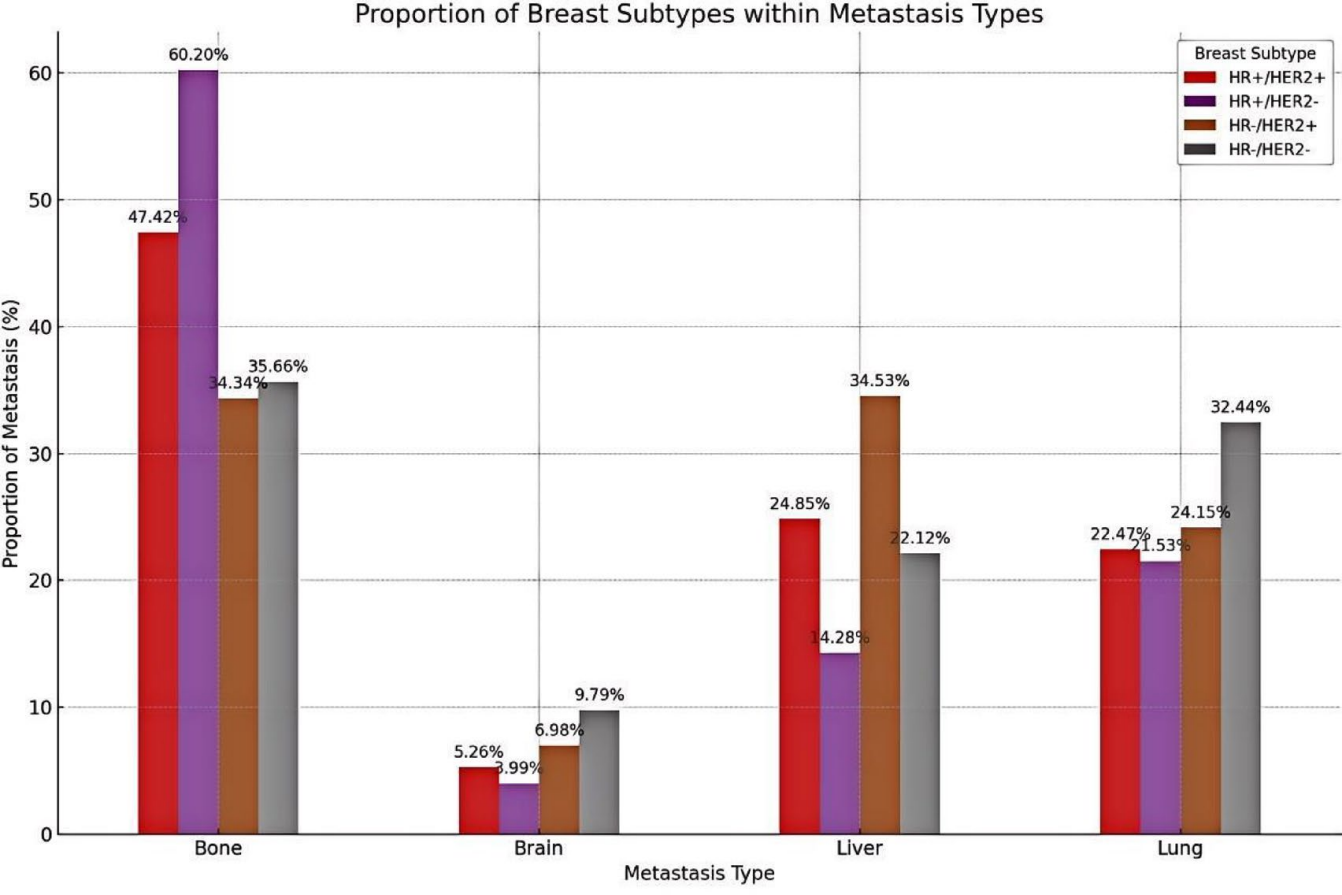
Distribution of breast cancer molecular subtypes across four single-organ metastatic sites. Stacked bar chart showing the relative composition of four molecular subtypes—HR+/HER2−, HR+/HER2+, HR−/HER2+, and HR−/HER2−—within each single-organ metastasis type (bone, liver, lung, brain).

The distribution of distant metastatic sites differed significantly across molecular subtypes. As illustrated in Fig. 3, among patients within each molecular subtype who exhibited single-organ metastasis, HR+/HER2 − tumors were predominantly associated with bone metastasis (accounting for 60.2% of single-site metastases in this subtype), whereas HR−/HER2 + and triple-negative tumors showed higher proportions of liver (34.5%) and brain (9.8%) metastases, respectively. Lung metastases were relatively evenly distributed, with HR−/HER2− (triple-negative) patients contributing slightly more cases (32.4%).

In contrast, Table 3 provides a complementary perspective by showing the proportion of patients within each molecular subtype who developed metastasis to specific organs, with the proportions calculated using the total number of patients within each subtype as the denominator. For example, 2.9% of patients with HR+/HER2 − tumors experienced bone metastases, whereas 4.8% of those with HR−/HER2 + tumors developed liver involvement. This distinction between organ-based and subtype-based analyses highlights the heterogeneous metastatic behavior of breast cancer and reinforces the importance of incorporating molecular subtype into risk stratification strategies at the time of diagnosis.

**Table 3:** Distribution of Organ-Specific metastasis across breast Cancer molecular subtypes at initial diagnosis (n and %).

Breast Subtype	Bone	Liver	Lung	Brain
HR+/HER2− (*n* = 152,434)	4371 (2.9%)	1037 (0.7%)	1563 (1.0%)	290 (0.2%)
HR+/HER2+ (*n* = 20,333)	972 (4.8%)	510 (2.5%)	461 (2.3%)	108 (0.5%)
HR−/HER2+ (*n* = 7,631)	364 (4.8%)	366 (4.8%)	256 (3.4%)	74 (1.0%)
HR−/HER2− (*n* = 20,058)	532 (2.6%)	330 (1.6%)	484 (2.4%)	146 (0.7%)

### Distribution of distant metastasis combinations

**Fig. 4:**
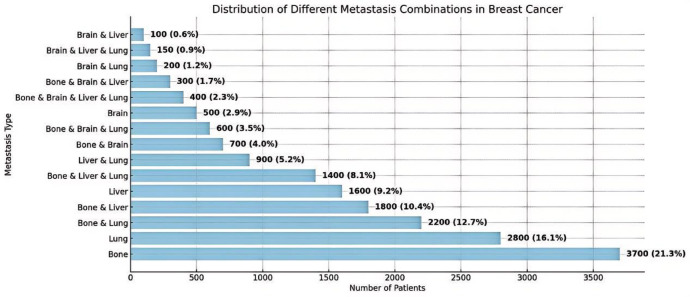
Distribution of single and combined distant metastasis types at diagnosis. Horizontal bar chart showing the frequency and proportion of patients with different single-site and multi-site metastasis combinations. Bone-only, lung-only, and bone + lung were the most common patterns.Molecular Subtypes Across Metastatic Combinations.

The distribution of distant metastasis combinations is presented in Fig. 4, with bone-only metastasis being the most frequent pattern (21.3%), followed by lung-only (16.1%) and bone + lung (12.7%).Other common combinations included bone + liver (10.4%) and liver-only metastasis (9.2%).Triple-site patterns such as bone + liver + lung (8.1%) and bone + brain + lung (3.5%) were less frequent.Quadruple-site metastasis involving all four organs (bone, liver, lung, and brain) was rare, observed in only 2.3% of patients.

### Molecular subtypes across metastatic combinations

**Fig. 5:**
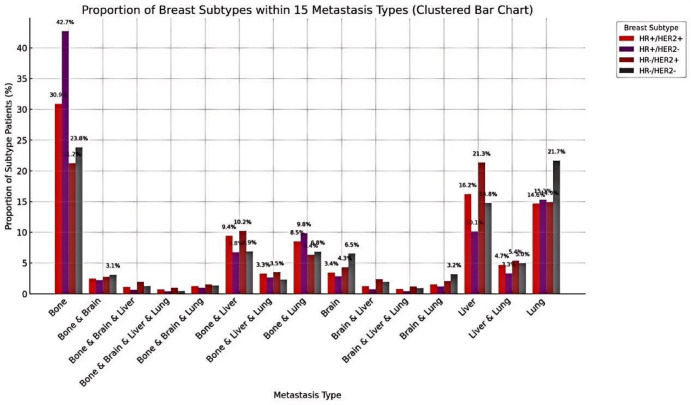
Proportion of molecular subtypes across 15 common metastasis combinations. The figure illustrates the relative proportions of four molecular subtypes (HR+/HER2−, HR+/HER2+, HR−/HER2+, and triple-negative) within each of the 15 most frequent distant metastatic patterns. For each metastatic combination, the sum of the subtype proportions equals 100%. The proportions were calculated based on the number of patients exhibiting each specific metastatic combination. HR+/HER2 − tumors were predominant in bone-only metastases, while brain- and liver-involved combinations showed higher frequencies of HR−/HER2 + and triple-negative subtypes.

The distribution of molecular subtypes across different metastatic combinations revealed distinct patterns. HR+/HER2 − tumors were predominantly associated with bone-only metastasis, accounting for 42.7% of cases within this group. HR−/HER2 + tumors were more frequently observed in liver-involved combinations, such as liver-only and liver + lung metastases, comprising up to 21.5%. Triple-negative breast cancers demonstrated a higher prevalence in lung-only (21.7%) and brain-involved metastatic patterns. In contrast, HR+/HER2 + subtypes were notably enriched in multi-organ combinations involving bone, liver, and lung, representing 16.8% of cases.(Figure 5).

### Survival outcomes based on metastatic patterns

**Fig. 6:**
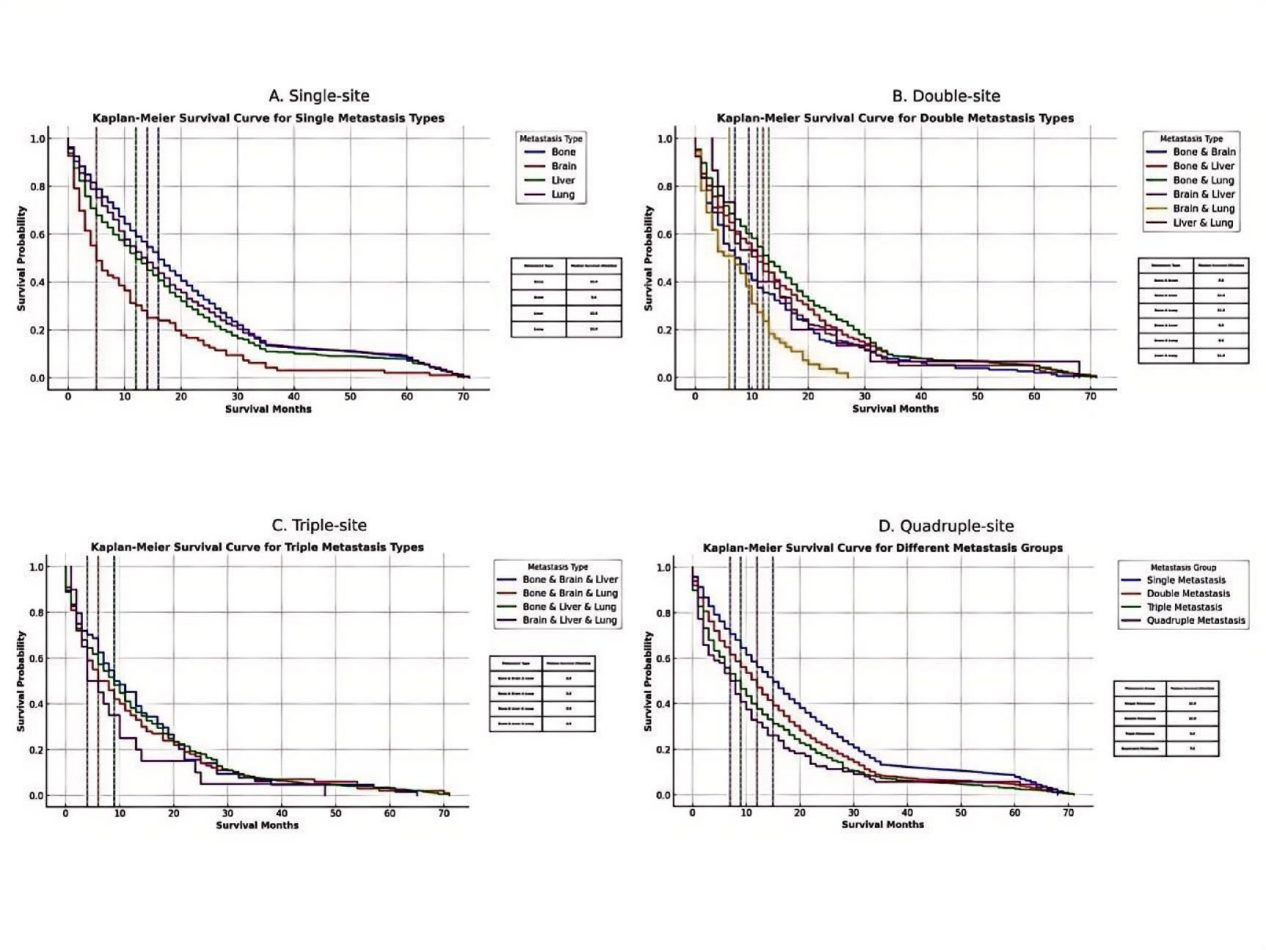
Kaplan-Meier survival analysis by metastatic pattern and burden. (**A**) Single-site; (**B**) Dual-site; (**C**) Triple-site; and (**D**) Quadruple-site metastasis. Median OS (overall survival) declined with increasing metastatic burden. Note: The survival curve for non-metastatic (M0) patients was excluded for visual clarity, as their substantially longer median survival (54.0 months; 95% CI: 53.5–54.6 months) would have compressed the time scale and impaired the visibility of differences among the metastatic subgroups.

Survival outcomes varied significantly by the number and pattern of metastatic sites. As shown in Fig. 6, patients with a single-site metastasis had the most favorable outcomes (Fig. 6A), with median OS of 16.0 months for bone, 14.0 for lung, 12.0 for liver, and 5.0 for brain.

Among dual-site metastases (Fig. 6B), the combinations of bone + lung (13.0 months), bone + liver (12.0), and liver + lung (11.0) showed the longest survival, while brain-involved combinations, such as brain + lung (6.0 months), had poorer prognoses.

In triple-site combinations (Fig. 6C), bone + liver + lung and bone + brain + liver had better outcomes (9.0 months), while liver + lung + brain had the shortest (4.0 months).

A consistent trend of decreasing survival was observed with increasing metastatic burden (Fig. 6D): 15.0 months for single-site, 12.0 for double-site, 9.0 for triple-site, and 7.0 months for quadruple-site metastasis.

To facilitate meaningful visual comparison across metastatic patterns, the survival curve for non-metastatic (M0) patients was not included in Fig. 6. This is because their median overall survival (54.0 months; 95% CI: 53.5–54.6 months) was substantially longer than that of any metastatic group, which would have compressed the time axis and obscured the differences among the M1 subgroups. Nevertheless, this survival estimate for M0 patients is reported here as a reference benchmark.

Detailed frequencies and corresponding median overall survival (OS) times for each metastatic combination are summarized in Table 4, arranged in descending order of median OS. Bone-only metastasis was the most common pattern, observed in 3,602 patients (1.8% of the total study population), and was associated with the longest median survival of 16.0 months. In contrast, metastatic patterns involving brain metastasis, particularly triple- and quadruple-organ combinations, were rare but exhibited the poorest outcomes, with median survival times as low as 4.0 months.

**Table 4:** Distribution and median survival of distant metastasis combinations in breast cancer, sorted by descending median overall survival. Patterns involving one or two metastatic sites are listed on the left; combinations involving three or more sites are listed on the right.

Pattern (1–2 Organs)	Frequency (*n*, %)	Median OS (mo)	Pattern (≥ 3 Organs)	Frequency (*n*, %)	Median OS (mo)
Bone only	3602 (1.80%)	16.0	Bone + Liver + Brain	65 (0.03%)	9.0
Lung only	937 (0.47%)	14.0	Bone + Liver + Lung	378 (0.19%)	9.0
Bone + Lung	919 (0.46%)	13.0	All four sites	89 (0.04%)	7.0
Bone + Liver	739 (0.37%)	12.0	Bone + Lung + Brain	101 (0.05%)	6.0
Liver only	673 (0.34%)	12.0	Liver + Lung + Brain	21 (0.01%)	4.0
Liver + Lung	181 (0.09%)	11.0			
Liver + Brain	16 (0.01%)	9.5			
Bone + Brain	153 (0.08%)	7.0			
Lung + Brain	56 (0.03%)	6.0			
Brain only	97 (0.05%)	5.0			

### Survival outcomes by metastatic burden

Overall survival decreased progressively with increasing metastatic burden. As shown in Fig. 6D, patients with a single metastatic site had a median survival of 15.0 months, compared with 12.0 months for double-organ metastases, 8.0 months for triple-organ metastases, and 7.0 months for quadruple-organ metastases. The detailed distribution of patient numbers, corresponding population percentages, and median survival times across each metastatic burden group is summarized in Table 5. Specifically, single-site and double-site metastases accounted for 2.73% and 1.06% of the total study population, respectively, while triple-site and quadruple-site metastases were much rarer, comprising only 0.29% and 0.04%.

**Table 5:** Survival outcomes by number of distant metastatic sites (metastatic burden) in breast cancer patients.

Burden (1–2 sites)	*n* (%)	Median OS (mo)	Burden (3–4 sites)	*n* (%)	Median OS (mo)
Single-site	5476 (2.73%)	15.0	Triple-site	589 (0.29%)	8.0
Double-site	2133 (1.06%)	12.0	Quadruple-site	89 (0.04%)	7.0

## Discussion

In this large-scale, population-based study utilizing the SEER database, we comprehensively analyzed the relationships between clinicopathological features and the risk, patterns, and survival outcomes of distant metastasis in breast cancer. Our findings reaffirm several established metastatic trends while also providing new insights into underexplored areas such as multi-organ spread, primary tumor location, and their interaction with molecular subtypes.

### Molecular subtypes and organotropism

Our results confirmed the strong organ-specific metastatic tendencies associated with molecular subtypes. HR+/HER2 − tumors were most frequently associated with bone-only metastases (60.2%), consistent with Smid et al.^3^, who found bone to be the preferred metastatic site for luminal A tumors. In contrast, HER2-enriched (HR−/HER2+) tumors in our cohort exhibited similar proportions of liver (34.5%) and bone (34.3%) metastases. While liver involvement was relatively more common compared to other subtypes, it is noteworthy that bone metastases were nearly equally frequent in this group. This balanced distribution suggests that both liver and bone are preferential metastatic targets for HR−/HER2 + tumors. Similar trends were observed by Kennecke et al.^2^, who reported liver involvement in 32% of HER2-positive metastatic patients.

Brain metastases at diagnosis were mainly observed in triple-negative (9.8%) and HER2-positive (7.0%) subtypes. Although the overall incidence of brain metastasis in the study population was low (0.3%), these two subtypes were overrepresented among cases with brain involvement. This pattern is consistent with previous reports. Niikura et al.^11^ found brain metastases in 34.2% of HER2-positive and 22.5% of triple-negative patients, compared to 8.6% in HR+/HER2 − cases. Lin et al.^5^ reported a 46% cumulative incidence in patients with metastatic TNBC. While those studies assessed brain metastasis during disease progression, the current findings highlight a similar subtype distribution at the time of initial diagnosis.

### Mechanistic insights on organotropism in molecular subtypes

The organ-specific metastatic behavior observed in our study may be partially explained by intrinsic molecular and cellular properties of each breast cancer subtype. For example, triple-negative breast cancers (TNBC) exhibit enhanced epithelial-mesenchymal transition (EMT), increased expression of SOX2 and CXCR4, and an ability to cross the blood–brain barrier, contributing to their high affinity for brain tissue^12^. HER2-positive tumors, on the other hand, are known to upregulate MMP-9 and VEGF, facilitating liver and lung metastasis via angiogenesis and extracellular matrix degradation^13^. Conversely, luminal A subtypes (HR+/HER2−) tend to express bone-homing receptors such as CXCL12/CXCR4, favoring osseous metastasis^14^. These molecular mechanisms offer biological plausibility to the organotropism trends observed in our population-based analysis.

### Multi-organ metastasis and prognosis

The current study demonstrated a clear inverse relationship between metastatic burden and overall survival (OS). Median OS decreased from 15.0 months in single-organ metastasis to 7.0 months in patients with metastases to all four sites. This pattern aligns with findings by Zhang et al.^8^, who reported a reduction in OS from 18.5 months in single-site to 7.2 months in multi-site metastasis using SEER data. In particular, brain-involved combinations such as brain + liver + lung were associated with the worst prognosis (median OS 4.0 months), consistent with the results of Arvold et al.^10^, who identified CNS involvement as a key indicator of poor outcome regardless of subtype.These findings are in line with Gu et al.^15^, who analyzed 1,888 patients with de novo stage IV triple-negative breast cancer and found that brain metastasis conferred the worst prognosis. Compared to brain metastases, patients with bone (HR = 0.516), lung (HR = 0.500), and liver metastases (HR = 0.670) had significantly better overall survival. This reinforces the high lethality of brain-involved metastatic patterns observed in our cohort.

### Comparison with prior studies on Multi-organ metastasis

Our study extends the findings of Zhang et al. who reported the prevalence and prognosis of single and dual metastatic sites using SEER data^8^. While prior analyses focused primarily on isolated or paired metastases, we profiled up to 15 metastatic combinations, including triple and quadruple organ involvement. Notably, we found that liver + lung + brain metastases were associated with the shortest survival (4.0 months) in our cohort, which was shorter than the survival reported by Jin et al.^16^. (7.0 months) and slightly lower than the median survival observed by Gu et al. among TNBC patients with central nervous system (CNS) involvement (5.2 months)^15^.This difference may be partly attributed to temporal variations in treatment practices. Our dataset includes patients diagnosed during earlier periods when systemic therapies, targeted agents, and immunotherapies were less widely available, Differences in molecular subtype composition and diagnostic timing may also have contributed to the observed discrepancies. Furthermore, the relative frequency of bone + lung (12.7%) and bone + liver (10.4%) combinations in our cohort supports the concept of dual organ preference in osseous–visceral patterns, as previously observed by Wu et al.^17^.

Although distant metastases in breast cancer have been widely studied, few investigations have detailed the distribution patterns of specific metastatic site combinations. In this cohort, bone-only metastasis was the most frequent (21.3%), followed by lung (16.1%) and liver (12.7%). Frequent dual-organ combinations included bone + liver (10.4%) and bone + lung (9.2%), suggesting a potential preference for osseous–visceral co-involvement. These trends are in line with Zhang et al.^8^ who reported similar distributions, including 22% for bone-only and 9.9% for bone + liver metastases. Notably, only 0.9% of patients had all four metastatic sites involved, indicating the rarity yet lethality of extensive multi-organ spread.

To date, only limited studies have explored the prognostic implications of specific multi-organ combinations. Jin et al. found that patients with brain-involved patterns, such as brain + liver + lung, exhibited the shortest survival, with a median OS of 7 months—closely reflecting the 4–7 month range observed in our cohort. By providing detailed frequencies and outcomes for 15 distinct metastatic combinations at diagnosis, the present study expands upon previous findings and offers valuable real-world evidence for individualized surveillance and prognostic assessment.

### Age and metastatic risk

Age was found to be a significant factor associated with site-specific metastasis in the present study, with patients aged ≤ 40 years exhibiting increased risks of bone (OR = 1.31) and liver metastases (OR = 1.55) compared to the 41–60-year reference group. These findings are in line with Wu et al.^17^, who reported that 6.1% of patients under 40 presented with M1 disease at diagnosis, versus only 1.8% in those aged ≥ 65. Similarly, Liu et al.^18^ found that liver metastases were significantly more common in patients younger than 50, suggesting a predilection for visceral spread in younger age groups. This trend may be partly explained by the higher prevalence of biologically aggressive subtypes, such as HER2-positive and triple-negative breast cancers, among younger patients. These observations support the need for age-specific screening and staging protocols to better identify high-risk metastatic patterns in younger populations.

### Tumor location and metastatic potential

In this study, tumors located in the axillary tail (C50.6) and unspecified regions (C50.9) were associated with a higher risk of distant metastasis compared to those in the upper-outer quadrant. This observation may be related to differences in lymphatic drainage pathways, limited breast tissue in these regions, or delayed detection due to less typical tumor presentation. Although direct comparative studies are limited, prior research has suggested that tumors in anatomically less common locations, such as the axillary tail, may exhibit earlier regional or distant spread due to their unique anatomical and lymphatic characteristics^19^.

### Histological grade and spread pattern

Higher histological grade was associated with a broader metastatic spectrum in this cohort. Grade III tumors exhibited more frequent visceral involvement, particularly in the liver and lungs, while Grade I tumors were more commonly linked to bone-only metastasis. These findings are consistent with Zhang et al.^8^, who reported distant metastases in 5.7% of Grade III versus 1.1% of Grade I tumors, with a corresponding decrease in median OS from 17.6 to 8.5 months. Similarly, Li et al.^20^ and Wang et al.^21^ found that Grade III breast cancers were more likely to metastasize to visceral organs, including the brain, and were associated with significantly worse survival outcomes. These results underscore the prognostic relevance of histological grade in determining both metastatic behavior and overall survival.

### Pathological classification

While our study did not find statistically significant differences in metastasis between ductal and lobular carcinoma overall, lobular carcinoma showed a trend toward increased bone-only spread. Arpino et al.^22^ and Cristofanilli et al.^23^ previously showed that invasive lobular carcinoma (ILC) tends to metastasize to bone, peritoneum, and gastrointestinal sites, while invasive ductal carcinoma (IDC) is more prone to liver and lung involvement. Our study supports this distinction and highlights the need to consider tumor type in follow-up imaging plans.

### Study strengths and limitations

The primary strengths of this study include its large sample size (*n* = 200,558), contemporary data range (2014–2023), and the use of real-world, population-based data from the SEER program. Unlike prior studies that primarily focused on isolated metastatic sites or single molecular subtypes, our analysis comprehensively evaluated 15 distinct metastatic combinations across four major organ systems, including rarely reported triple- and quadruple-organ metastases. Brain-involved metastatic patterns were systematically profiled, highlighting their disproportionately poor survival outcomes. Moreover, the incorporation of clinicopathological variables—such as age, tumor location, histological grade, and molecular subtype—enabled a multifactorial understanding of metastatic behavior and prognosis.

Nevertheless, several limitations should be acknowledged. First, the SEER database captures only metastases present at initial diagnosis, excluding metachronous or treatment-emergent metastases. Second, treatment-related information, including chemotherapy regimens, targeted therapies (e.g., anti-HER2 agents), and endocrine therapy, was unavailable and may have influenced survival outcomes. Third, uncommon metastatic sites, such as the peritoneum or skin, were not recorded, thereby limiting a comprehensive analysis of the full metastatic spectrum. Fourth, as the data span nearly a decade, temporal changes in diagnostic techniques and treatment advances may have impacted survival outcomes and should be considered when interpreting the results.Fifth, the SEER database does not include time to recurrence or duration from recurrence to death; therefore, disease-free interval (DFI) and post-recurrence survival could not be assessed in this study. As such, we were unable to analyze the prognostic differences between early recurrence (e.g., within two years) and late recurrence (e.g., after five or ten years), which have been recognized as important clinical distinctions in breast cancer outcomes. Sixth, morphological characteristics of metastatic lesions (e.g., size, growth pattern, imaging appearance) could not be evaluated, as SEER does not capture such data. Therefore, differences in morphology among various organ metastases were not analyzed. Seventh, although SEER provides a large population-based dataset, the cases are collected from specific geographic regions in the United States. Thus, the findings may not be fully generalizable to populations outside the SEER catchment areas or to international settings. Finally, the dataset was imbalanced across different metastatic subgroups, which may have affected the weighting of logistic regression models and the stability of adjusted odds ratios. While the large sample size mitigates some of this effect, this limitation should be considered when interpreting the results.

## Conclusion

This large-scale, population-based study of over 200,000 breast cancer patients from the SEER database provides a comprehensive evaluation of the clinicopathological factors associated with site-specific and multi-organ distant metastases. We demonstrate that distinct metastatic patterns are closely linked to molecular subtypes: HR+/HER2 − tumors predominantly metastasize to bone, whereas HER2-positive and triple-negative subtypes exhibit a greater propensity for visceral and brain involvement. Importantly, multi-organ metastases—particularly those involving the brain—are associated with markedly poorer survival outcomes.

In addition, we identify younger age, higher histological grade, and tumor location in the axillary tail or unspecified regions as independent risk factors for more aggressive metastatic behavior. Grade III tumors not only display broader metastatic distributions but are also correlated with significantly inferior survival, reinforcing their biological aggressiveness.

To our knowledge, this is the first study to systematically characterize 15 distinct metastatic site combinations and their associated survival outcomes within a contemporary, molecularly stratified breast cancer cohort. These findings offer important insights that may inform the development of subtype-specific surveillance strategies, personalized risk assessment tools, and individualized prognostic models.

Future research should further elucidate the molecular mechanisms underlying metastatic organotropism and leverage emerging technologies, such as artificial intelligence–based predictive frameworks, to enhance early identification and targeted management of high-risk metastatic patterns.

## Data Availability

The datasets generated and/or analysed during the current study are available in the SEER repository: https://seer.cancer.gov/data/. Access to the SEER database requires users to register and sign a data use agreement. The authors accessed the SEER*Stat 8.4.2 research data (November 2023 submission).
